# Lobomycosis in Inshore and Estuarine Dolphins

**DOI:** 10.3201/eid1504.080955

**Published:** 2009-04

**Authors:** Alberto Enrique Paniz-Mondolfi, Lilian Sander-Hoffmann

**Affiliations:** Columbia University College of Physicians and Surgeons, New York, New York, USA (A.E. Paniz-Mondolfi); Instituto de Biomedicina, Caracas, Venezuela (A.E. Paniz-Mondolfi, L.Sander-Hoffmann); Universidade Federal do Rio Grande do Sul, Porte Alegre, Brazil (L. Sander-Hoffmann)

**Keywords:** Zoonoses, lobomycosis, dolphins, inshore, Tursiops truncatus, Sotalia guianensis, letter

**To the Editor:** Lobomycosis is a chronic dermal infectious disease affecting humans and some species of dolphins but not, to date, freshwater dolphins. Because this disease is still considered rare despite the increasing number of reported cases in humans and cetaceans, clinical and epidemiologic information must be accurately reported to help clarify many of the unknown aspects of this disease.

We address this point because after carefully reading the excellent report by Elsayed et al. on the first human case of lobomycosis in Canada, we noticed that the authors describe the natural disease as occurring in humans and marine and freshwater dolphins only (*1*). However, this information is only partially correct because to date lobomycosis has not been described in freshwater dolphins. What is more worrisome is that this information is beginning to be referenced in other published articles (*2*). So far, lobomycosis has been confirmed in 2 species of inshore and estuarine Delphinidae: 1) the common bottlenose dolphin (*Tursiops truncatus*) from Brazil, the Atlantic coast of the United States, and Europe and 2) the Guiana dolphin (*Sotalia guianensis*) from the Surinam River estuary (*3*–*8*).

The fact that lobomycosis is endemic in humans in the Amazon basin could logically raise the suspicion that other animal species in this area may act as reservoirs or even be affected by the disease. However, the infection has never, to our knowledge, been reported in botos (*Inia geoffrensis*) or tucuxis (*Sotalia fluviatilis*) from the Amazon and Orinoco Rivers. Preliminary field studies, like the one carried out by da Silva et al., failed to demonstrate the disease in any of the 385 live-captured *I. geoffrensis* boto specimens from the Mamirauá Reserve in the central Amazon region of Brazil (*9*); similarly, our observational studies in the Venezuelan Orinoco River failed to detect the disease. On the other hand, despite the absence of indigenous cases of lobomycosis in humans reported in the United States, the disease is endemic in dolphins from the Indian River Lagoon in Florida (*7*), suggesting that no apparent epidemiologic link may exist between humans and cetaceans. Unfortunately, the etiologic agent of lobomycosis, *Lacazia loboi* (Figure), has not been cultured in vitro (*10*) despite exhaustive attempts, making its isolation from probable and suspected environmental sources impossible.

**Figure:**
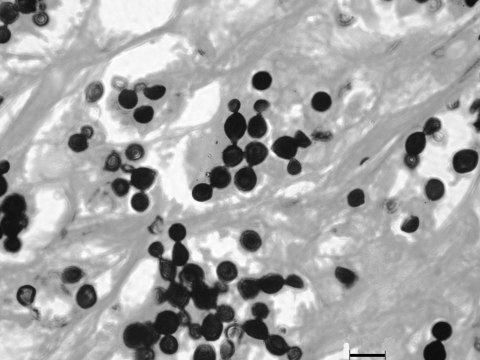
Grocott methamine silver–stained section from a skin biopsy specimen of a bottlenose dolphin (*Tursiops truncatus*) showing abundant *Lacazia loboi* yeast cells individually and in chains connected by thin tubular bridges. Magnification ×400.

Dolphin-to-human transmission of lobomycosis has been reported only 1 time; the case-patient was an aquarium attendant who had had close physical contact with an affected dolphin (*5*). However, because the possibility of zoonotic transmission of this disease remains latent and because many pathologic and clinical aspects of the disease remain poorly understood, it is imperative to clarify these ecological concepts. Up-to-date molecular epidemiology studies to compare the strains affecting humans and dolphins and their possible phylogenetic relationship are needed.
